# The Relationship between Quality of Life and Physical Activity, Worry, Depression, and Insomnia in Pregnant Women

**Published:** 2020-04

**Authors:** Adham Davoud, Malek Abazari

**Affiliations:** 1 Department of Public Health, School of Health, Ardabil University of Medical Sciences, Ardabil, Iran.; 2 Department of Public Health, School of Public Health, Ardabil University of Medical Sciences, Ardabil, Iran.

**Keywords:** *Depression*, *Insomnia*, *Pregnant Women*, *Physical Activity*, *Quality of Life*, *Worry*

## Abstract

**Objective:** Physical activity (PA), insomnia, depression, and worry were the key factors affecting pregnant women’s quality of life (QoL). The present study aimed to determine quality of life and its relationship with physical activity, insomnia, depression, and worry in pregnant women.

**Method** **:** This was an observational cross sectional study, conducted among 256 healthy pregnant women using 5 questionnaires: WHOQOL-brief (WHO Quality of Life Questionnaire, brief version, ISI (Insomnia Severity Index), PSWQ (Penn State Worry Questionnaire), ZSRDS (Zung Self-Rating depression Scale), and Pregnancy Physical Activity Questionnaire (PPAQ).

**Results: **There was a significant relationship between general QoL, insomnia, and worry with educational background, number of children, and occupation (P < 0.05). Depression had a significant relationship with occupation (P < 0.05). PA did not have a significant relationship with demographic information. However, insomnia had a significantly negative relationship with general QoL, general health, and psychological health (P < 0.05). The worry variable had also a significant negative relationship with general QoL, general health, and physiological health (P < 0.05). Depression had a significant negative relationship with general health, physical health, and psychological health (P < 0.05). There was no significant relationship between physical activities in pregnancy with QoL, depression, worry, and insomnia.

**Conclusion: **Women need to be informed about the necessity of controlling and reducing insomnia, worry, and depression to have a higher QoL. PA declined during the second and third trimester of pregnancy. However, PA in pregnancy can positively impact general QoL.

Pregnancy and childbirth are natural events in women's life cycle. Yet, although pregnancy is considered as a natural activity done by women, it is a highly stressful experience for them. This experience makes pregnant women undergo many psychological and physical changes (1). Lack of ample knowledge about appropriate pregnancy care, lack of PA, and gaining extra weight cause anxiety, difficulty, and unexpected negative consequences in pregnant women (2). Pregnancy is a period in which women experience psychological and physiological changes in a way that they may face some specific problems and tensions, such as continuous nausea, fatigue, and sudden pains. These problems can heavily affect women's capabilities for doing their ordinary, routine activities in life (3). 

Women who experience anxiety in their pregnancy are likely to suffer from emotional imbalance even after their pregnancy and throughout the rest of their life (4). 

One of the important factors in woman's general health is QoL (5). QoL includes various aspects of one's health, physical well-being, and sociopsychological wellbeing; QoL is a direct result of general health that is considered to be important in evaluating pregnancy treatments and pre pregnancy cares (6). Few studies have investigated pregnant women's QoL, as most of them have focused only on the QoL in patients with high blood pressure and diabetes (7, 8). Regular physical activity during pregnancy lowers the risk of pregnancy diabetes, preeclampsia, and pregnancy overweight (9).

The latest instruction by the American Colleges of Obstetricians and Gynecologists (ACOG) recommends that women without physical and child delivery problems should have physical activity at least once a week for 30 minutes (10). There are a few studies on physical activity of pregnant women and the majority of them are limited to the following topics: (1) measuring physical activity during leisure times; (2) intense physical activity; (3) using the tools that cannot measure frequency, intensity, and time period of activity; and (4) focusing on physical activity after child delivery. In addition, there are even fewer studies on the relationship between physical activity and quality of life in pregnant women (11-15). Thus, it is necessary to conduct more studies on physical activity of pregnant women to provide more information for future studies and to improve physical activity in pregnant women. 

Insomnia during pregnancy has a relationship with increase in the risk of cardiovascular diseases, hypertension, diabetes, neurological problems, and psychological disorder. In addition, these problems may result in an increase in BMI after pregnancy (16, 17). A decrease in sleep time to less than 6 hours may lead to embryo development disorders and prolonged child delivery (18). Prevalence of insomnia in European countries in pregnant women ranges from 52% to 61% (19, 20). There are a few studies on the prevalence of insomnia and its relationship with quality of life in pregnant women in Iran. Therefore, there is a need for more studies on insomnia in pregnant women. 

One of the factors that plays a key role in the general health of pregnant women is pregnancy-related worry, which is a specific type of worry that refers to the motherhood fears and concerns during pregnancy and covers worries about fetus health, physical symptoms, child bearing, interpersonal relationships, and child birth (21). Mental pressures and worries of pregnancy affect the body’s physiological performance directly and results in a lower general health in pregnant women. On the other hand, unwanted events in life, concerns about child birth, and BMI affect general health of women. These women may be even faced with psychological problems like depression and anxiety (22). Studies have shown that worry can degrade quality of life and general health (23, 24). Still, there has been a paucity of studies on the prevalence of worry in pregnant women and its relationship with quality of life. Therefore, there is a need for more studies in this area. 

Another key factor about the general health of pregnant women is their depression. Studies have shown that depressed women do not have mental health and are vulnerable to variety of tensions. According to these studies, pregnancy is a critical period (25, 26). Findings have shown that depression of pregnant women may be due to the lack of physical activity. Studies have also shown that low mental health and physical activity may lead to pregnancy-related health problems (27, 28). 

There have been a very few studies in Iran on the relationship between the quality of life and physical activity in pregnant women. There is also a paucity of studies on insomnia, worry, and depression in pregnant women. Therefore, the present study was an attempt to elaborate on the relationship of physical activity, insomnia, worry, and depression with quality of life in pregnant women. In fact, this study aimed to achieve 3 goals. The scores of the quality of life, depression, worry, insomnia, and physical activity during pregnancy were determined during the first 3 months of pregnancy. Then, the relationship of the quality of life with depression, worry, insomnia, and physical activity during pregnancy was examined. In the end, the effects of depression, worry, insomnia, and physical activity during pregnancy on the subscales of quality of life in pregnant women were determined.

## Materials and Methods

***Study Design and Participants***

This study was performed as a retrospective cross sectional study and the study population were pregnant women visiting health centers in Ardabil. Given that age younger than 18 years during pregnancy is considered as risky pregnancy and that clinical and psychological diseases or unpleasant events in life are the sources of worry, anxiety, and depression, inclusion criteria in this study were as follow: being older than 18 years during pregnancy, having Iranian nationality, having a Muslim faith, living in Ardabil, living with the spouse, and willingness to participate. Exclusion criteria were pregnancy with embryonic disorders and multiple fetus, overnight work shift, history of clinical or psychological diseases (self-statement), familial problems (self-statement), history of sterility, experiencing unpleasant life experience over the past 9 months (loss of blood relatives), financial problems, and losing job (mother or the father based on self-statement).

***Sample Size and Sampling***

Based on Zarei et al's article (29), the sample size, with SD of 0.3 for QoL, confidence interval of 0.05, d of 0/15σ, and effect size of 1.5, was calculated as 256. The sampling method was 2-level clustering in which 5 centers were first selected randomly from health centers in Ardabil; then, 56 pregnant women were selected from each center based on availability for interviews. Participants were given general information about the research and confidentiality of the identities. Since the interview involved many questions, some women were unwilling to participate (28 Cases), and only those women participated in the study who were willingly volunteered and consented to answer the questions; thus, convenience sampling method was used. 

***Data Collection***

The required data were collected by interviews and completing questionnaires. Six trained interviewers conducted the interviews, and the questionnaires were filled in immediately after the interviews by participants. Aside from demographic information, the following questionnaires were used:

**QOL Questionnaire:** The WHOQOL-brief (The World Health Organization QOL) Questionnaire as a truncated version of the WHOQOL-100 questionnaire was employed. This is a valid instrument to measure the patients' QoL. This self-administered instrument includes 1 section on QoL (1 question) and general health (2 questions) and 1 section on satisfaction from QoL was divided to 4 categories: physical health (7 questions), physiological health (6 questions), social relationships (3 questions), and environmental health (8 questions). Each item is based on a 4-point Likert-scale categorization. After the scores belonging to each section are calculated, the total standard score is calculated within a range from 0 to 100, in which 100 shows the highest QoL possible. The validity and reliability of the questionnaire have been supported by studies in Iran and other countries, and the reliability of the scale in Iran was confirmed by Montazeri et al. Internal consistency of the questionnaire was higher than 0.7 (the Cronbach’s alpha coefficients ranging from 0.77 to 0.90) (28, 30, 31). 

**Insomnia Severity Index Questionnaire:** This is a self-administered instrument based on DSMIV diagnosis criteria for insomnia, which measures the severity of insomnia in the last 2 weeks. The questionnaire comprises 7 questions that measure sleep duration, early wake-up (either at night or in the morning), satisfaction from the current sleep pattern, disorder in daily activities, and deterioration of QoL. Each item is based on a 5-point Likert-scale categorization. The final total score ranges from 0 to 28 with 0-7 showing lack of clinical insomnia, 8-14 on the clinical threshold, 15-21 moderate insomnia and 22-28 severe insomnia. Validity and reliability of the questionnaire have been supported by studies in Iran and other countries, and the reliability of the scale in Iran was confirmed by Yazdi et al. Internal consistency of the questionnaire was higher than 0.7 (the Cronbach’s alpha coefficients was 0.87) (32-34).

**Worry Questionnaire:** This is a self-administered questionnaire validated by TJ Meyer designed to diagnose intense, chronic, uncontrollable worry in patients. This questionnaire includes 16 five-point questions. The final score ranges from 16 to 80. A score above 44 shows a significant clinical level of worry (44-62 shows a moderate level of worry and 63-80 a high level of worry). Validity and reliability of the questionnaire have been supported by studies in Iran and other countries, and the reliability of the scale in Iran was confirmed by Salarifar et al. Internal consistency of the questionnaire was higher than 0.7 (the Cronbach’s alpha coefficients was 0.83) (35, 36).

**Depression Scale Questionnaire:** This questionnaire was originally published by Zung based on a scientific definition in 1965. The Zung self-administered depression scale was designed to gauge the severity of depression. Despite being short and simple, this scale includes all aspects of depression and has been used in many studies focusing on depression. The depression scale measures different aspects of emotional, cognitive, behavioral, and psychological depression. It includes 20 questions on various aspects of temper (depression severity) and is scored from 1 to 4, with 10 negative points and 10 positive points. Individuals are required to answer the questions in a 4-point Likert-scale based on their experience in the given situations. A score below 50 shows a normal temper without any psychological disorders, a score of 50-59 shows mild-to-moderate depression, a score of 60-69 shows moderate-to-severe depression, and a score beyond 70 shows a severe depression. Validity and reliability of the questionnaire have been supported by studies in Iran and other countries, and the reliability of the scale in Iran was confirmed by Rashedi et al. Internal consistency of the questionnaire was higher than 0.7 (the Cronbach’s alpha coefficients ranging from 0.74 to 0.94) (37, 38). 

**PA Questionnaire**: The standard PPAQ questionnaire is related to PA in pregnancy. This questionnaire is divided to 4 question groups, including home activities (16 questions), commuting activities (3 questions), work activities (5 questions), and leisure/sports activities (8 questions). The severity of activities is measured based on the MET scale, which is a unit for the metabolic exit while performing physical activities (each MET equals the use of 3.5 mL oxygen per kilogram body weight). To measure the severity of PA, the met amount of each activity is multiplied by the time spent per day. Then, the severity of PA is calculated by the sum of all activities per day. In total, an activity with a MET lower than 1.5 is considered as inactivity, 1.5-3 as light activity, 3-6 as moderate activity, and above 6 as severe activity. Validity and reliability of the questionnaire have been supported by studies in Iran and other countries, and the reliability of the scale in Iran was confirmed by Bahadoran et al. Internal consistency of the questionnaire was higher than 0.7 (the Cronbach’s alpha coefficients ranging from 0.78 to 0.94) (39, 40). 

***Statistical Analysis***

Sociodemographic characteristics and health status have been described for the entire sample. The mean and standard deviation were calculated to describe quantitative variables. Frequency and percentage were calculated to describe the variables.

The Kolmogorov-Smirnov test was used to examine the normality of quantitative variables at the samples. One-way analysis of variance was used to compare continuous variables among groups (more than 2 categories). Kruskal-Wallis tests were used when normality of the distribution of the variables was not met.

Student tests were used to compare quantitative variables between the 2 groups. Also, Mann-Whitney tests were used when the normality of the distribution of the variables was not met. Fisher’s exact tests and chi-square tests were used to compare qualitative variables between the 2 or more groups.

Multivariate analysis (more than one response) was used to examine the effect of independent variables on the response variables.

The statistical software SPSS for Windows version 18.0 (Chicago, IL, USA) was used for statistical analysis. Significance level was set at p ≤ 0.05. 

## Results

***Demographic Information***

The mean (SD) of the participants was 29.31(4.98), and 69.9% of the participants had academic education and 75.8% had 1 or 2 children; 12.5% of them were working and the rest were housewives. Moreover, 71.9% of the participants were not satisfied with their family income (Table 1).

***Questionnaire Scores in Different Pregnancy Trimester Periods***

The results of the study showed that the score for QoL increased with the pregnancy months (P < 0.05), and the depression score in the last trimester was higher than in previous ones (P < 0.05). The level of insomnia and worry were not significantly related to the pregnancy trimesters (Table 2). Based on the results, 82.4% of the participants experienced different instances of insomnia, 80.8 suffered from worry, and 56.3% were not depressed at all (Table 3). 

The Relationship between QoL Subcategories and Demographic Information

There was a significant relationship between general QoL and educational background (P < 0.001), number of children (P < 0.001), and occupation (P = 0.019). Pregnant women who had 1 or 2 children and had only primary education and were housewives enjoyed a higher QoL. General health had a significant positive relationship with educational background as an increase in education level raised general health (p = 0.011). In addition, psychological health was negatively related to age and number of children, as an increase in the age of participants decreased their psychological health (r = -0.26, P = < 0.001), and those women who did not have any children had better psychological health (P = 0.048). 

***The Relationship between PPAQ, ZSDS, PSWQ, and ISI Subcategories and Demographic Information***

Insomnia had a significant relationship with educational background and number of children (P < 0.05). Women who had 1 or 2 children and had primary education gained a lower score of insomnia (P < 0.05). Similarly, worry had a significant relationship with educational background and number of children, as women who did not have any children and had secondary education or higher received a higher score in insomnia (P < 0.05). Depression had a significant relationship with occupation in a way that women who had an occupation also experienced higher levels of depression than housewives (P < 0.05). Nevertheless, PA did not have a significant relationship with demographic information (Table 4). 

***The Relationship between QoL and Insomnia, Depression, Worry, and PA in Pregnancy***

Insomnia had a significant negative relationship with general QoL (r = -0.336, P < 0.05), general health (r = -252, P < 0.05), physical health (r = -0.284, P < 0.05), and psychological health (r = -0.281, P < 0.05). On the contrary, insomnia did not have any significant relationships with social relationships and environmental health. The worry variable had also a significant negative relationship with general QoL (r = -0.395, P < 0.05), general health (r = -0.205, P < 0.05), physical health (r = -0.149, P < 0.05), and physiological health (r = -0.216, P < 0.05), but it did not have a relationship with social relationship and environmental health. Likewise, depression had a significant negative relationship with general health (r = -0.125, P < 0.05), physical health (r = -0.195, P < 0.05), and psychological health (r = -0.169, P < 0.05), but it did not have any relationships with general QoL, social relationships, and environmental health. Yet, the results of this study showed no significant relationship between physical activities in pregnancy and QoL, depression, worry, and insomnia.

***The Effects of Insomnia, Worry, Depression, and Physical Activities in Pregnancy on the Subcategories of QoL***

The results of multivariate regression showed that insomnia and worry variables had significant effects on general QoL, general health, physical health, and psychological health, but they did not have significant effects on social relationships and environmental health (P < 0.05). Furthermore, the effects of depression variable on general QoL, physical health, and psychological health were significant, but they were not significant on general health, social relationships, and environmental health (P < 0.05). The PA variable in pregnancy did not have any significant effects on the subcategories of general QoL (Table 5). 

## Discussion

This study is one of the scarce pieces of research that aimed to address the following issues: (a) calculating the score of the QoL, depression, worry, insomnia, and PA in pregnancy in pregnancy trimester periods; (b) investigating the relationship between QoL and depression, worry, insomnia, and PA in pregnancy; and (c) studying the effects of depression, worry, insomnia, and PA in pregnancy on the subcategories of QoL. 

The findings of this study demonstrate that general QoL, general health, and psychological health as 3 subcategories of QoL increased with the advancement in pregnancy months. This finding may show that pregnant women become more self-protective as they move forward in their pregnancy and health services are more available. As for other subcategories of QoL, no significant relationship was observed with pregnancy trimester periods. This finding is in line with the report from another study in which it was observed that QoL in the second and third trimesters was better than the first period (41). However, another study found no significant relationships between the QoL and any pregnancy trimester periods (42). 

This study found that general QoL as one subcategory of QoL had a significant negative relationship with educational background of participants, as pregnant women with lower education were more satisfied with their lives. This can be due to the increase of education level and expectations in life. In addition, housewives with children enjoyed a higher QoL than women who worked outside and did not have any children. One previous study reported that QoL was not significantly related to educational background, number of children, and having a job (42). Yet, another study found a significant negative relationship between educational background and QoL (43). 

Moreover, it was found that general health was higher in educated women. This shows that women with higher education are considerably more self-protective. In agreement with another study (42), this study revealed that pregnant women with children were more physically healthy than those who were experiencing pregnancy for the first time.

Depression, worry, and insomnia were not significantly related to the 3 trimesters of pregnancy. The reason for this finding may be that most of the participating women were suffering from depression, insomnia, and worry in a way that 80% of pregnant women had worries (62.4% moderate and 18.4 severe) and 73% were suffering from insomnia (43.8% weak, 29.3% moderate and 9.6 severe). Half of the participants also suffered from depression. Previous studies have shown that 35% of pregnant women suffer from worries, which is lower than the findings of this study (44). It has also been found in the literature that the depression level of pregnant women was 30.5% (42) and insomnia prevalence was 57%, without any noticeable change in pregnancy advancement (45). However, one study in Spain observed that insomnia was more severe in the third trimester than it was in the first and second counterparts: 44% for the first trimester, 46% for the second one, and 64% for the last one (46). The high prevalence of insomnia, depression, and worry in this study can be a result of dissatisfaction with the country's economic condition, occupational status, and income because 87.5% of women were unemployed and 71.9% were dissatisfied with their family's economic condition. Previous studies have revealed a negative relationship among worry, environmental health, and psychological health and demonstrated that an increase in the depression level may decrease all aspects of QoL; a high score of insomnia is also another factor that drastically lowers the QoL(26, 42, 47). The results of this study also showed that severe insomnia could decrease general QoL, general health, physical health, and psychological health, and that higher scores of worry and depression could deteriorate the level of general health, psychical health, and psychological health. 

PA in pregnancy is very important and can lead to better physical and psychological health (48). Earlier studies have observed that the amount of PA decreases until the 28th week of pregnancy, from the first trimester period to the second one or from the first to the last trimester period (49, 50). This study also reveals that the PA of pregnant women was lowered in the second and third pregnancy trimesters as compared to the first one. Lack of ample PA in pregnancy, especially in the second and third trimesters was the result of this false assumption that pregnant women should only take a rest, which leads to obesity (51). Research has demonstrated that having children and higher education are significantly related to having more PA in pregnancy (52, 53). Nevertheless, there was not a significant relationship between PA and demographic information in this study. Although previous studies showed that PA in pregnancy can lead to improved health and higher QoL in pregnant women (54, 55), this study found no significant relationship between PA in pregnancy and various subcategories of QoL. The reason for such a finding may be the highly prevalent inactivity in Iranian pregnant women as a study in Asia which used the same questionnaire found the mean of PA to be 150 (42), whereas it was 141 in this study in the second and third pregnancy trimester periods. The results of this study clearly showed the effects of depression, worry, and insomnia on different aspects of QoL. It was found that insomnia affected general QoL, general health, physical health, and psychological health, indicating that a decrease in depression could increase the 4 subcategories of QoL. Similarly, a decrease in the intensity of worry increased general QoL, general health, physical health, and psychological health altogether. 

As for the depression, the results revealed that a decrease in depression level could raise general QoL, physical health, and psychological health. The negative relationship between QoL and depression, insomnia, and worry has also been reported in previous studies (26, 42, 55). In addition, a positive relationship was found among depression, worry, and insomnia, as a change in any one of these variables caused the others to change, meaning that if a woman suffers from one of them, she probably experiences the other 2 as well; this finding also agrees with previous studies (56, 57).

**Table 1:** Participants’ Characteristics of the Study Population, Mean (sd) or Number (Percent)

**Variable**	**Total** **N=256**	**Trimester 1** **N=55**	**Trimester 2** **N=108**	**Trimester 3** **N=93**	**P-value**
Age(years)	30.18(5.74)	25.35(6.04)	27.82(2.87)	35.78(2.89)	<0.0001
Education^a^					<0.0001
Primary	27(10.5)	2(3.6)	1(0.9)	24(25.8)	
Secondary	50(19.5)	34(6108)	10(9.3)	6(6.5)	
University	179(69.9)	19(34.5)	97(89.8)	63(67.7)	
Number of previous deliveries					<0.0001
0	62(24.2)	36(65.5)	25(23.1)	1(1.1)	
1,2	194(75.8)	19(34.5)	83(76.9)	92(75.8)	
Job					0.003
Worker	32(12.5)	8(14.5)	5(4.6)	19(20.4)	
Housewives	224(87.5)	47(85.5)	1.3(95.4)	74(79.6)	
Sufficiency of income for expenses					0.2
Sufficient	72(27.1)	18(32.7)	24(22.2)	30(32.3)	
Insufficient	184(71.9)	37(67.3)	84(77.8)	63(67.7)	
Abortion					0.653
No	203(79.3)	46(83.6)	85(78.7)	72(77.4)	
Yes	53(20.7)	9(16.4)	23(21.3)	21(22.6)	

a Kruskal Wallis test

**Table 2:** Average Scores ± Sd for Insomnia, Worry, Depression, QOL and PA Domains (ISI, PSWQ, ZSDS, PPAQ, WHOQOL-Brief Questionnaires)

**Variable**	**Total** **N=256**	**Trimester 1** **N=55**	**Trimester 2** **N=108**	**Trimester 3** **N=93**	**P-value**
General QOL	70.93(16.2)	56.32(17.65)	67.47(5.58)	83.6(14.31)	<0.0001*
General health	71.61(18.57)	64.02(19.75)	71.83(17.32)	75.84(18.08)	0.001*
Physical health	65.33(16.41)	32.37(18.05)	63.61(15.48)	66.32(16.41)	0.307
Psychological health	60.68(17.66)	55.19(17.04)	59.94(15.8)	64.8(19.17)	0.005*
Social relationships	58.13(18.09)	55.66(18.44)	58.72(17.76)	58.9(18.33)	0.52
Environment	57.78(19.28)	59.79(17.78)	58.69(19.55)	55.53(19.8)	0.351
ISI	12.78(5.62)	13.10(5.32)	13.03(5.37)	12.31(6.1)	0.596
PSWQ	52.44(12.31)	54.4(15.47)	52.58(10.33)	51.11(12.31)	0.29
ZSDS	49.6(12.31)	48.91(12.78)	50.49(11.8)	48.97(12.65)	0.615
Activity	145.97(56.49)	162.77(62.04)	141.17(49.01)	141.97(59.80)	0.045
Household/care giving	76.75(39.90)	82.81(41.36)	77.11(27.69)	72.75(79.79)	0.333
Sports/exercise	5.33(3.43)	5.62(4.16)	5.49(3.37)	4.97(3.02)	0.441
Transportation	20.74(12.85)	19.82(12.74)	20.86(11.95)	21.14(13.97)	0.826
Occupational^a^	N=32 48.00(21.53)	N=8 43.6(24.17)	N=5 55.13(26.03)	N=19 47.97(19.95)	0.623

QOL: quality of life, PA: Physical activity, ISI: Insomnia Severity Index, PSWQ: Penn State Worry Questionnaire, ZSDS: Zung Self-Rating Depression Scale, PPAQ: Pregnancy Physical activity questionnaire, WHOQOL: World Health Organization Quality Of Life

a Among working women and Kruskal-Wallis tests

**Table 3:** Categorized Results of the Study Population for Insomnia (ISI), Worry (PSWQ) and Depression (ZSDS)

**Variable**	**Total** **N=256**	**Trimester 1** **N=55**	**Trimester 2** **N=108**	**Trimester 3** **N=93**	**P-value**
ISI					0.132
Lack of clinically significant insomnia	45(17.6)	14(25.5)	14(13)	17(18.3)	
Subclinical insomnia	112(43.8)	18(32.7)	57(52.8)	37(39.8)	
Moderate clinical insomnia	75(29.3)	19(34.5)	29(26.9)	27(29)	
Severe clinical insomnia	24(9.4)	4(7.3)	8(7.8)	12(12.9)	
PSWQ					0.002*
No clinically significant level of worry	49(19.2)	5(9.1)	32(29.6)	12(13)	
Moderate level of worry	159(62.4)	42(76.4)	54(50)	63(68.5)	
High level of worry	47(18.4)	8(14.5)	22(20.4)	17(18.5)	
ZSDS					0.527
Absence of depression	144(56.3)	29(52.7)	64(59.3)	51(54.8)	
Mild depression	80(31.3)	20(36.4)	32(29.6)	28(30.1)	
Moderate depression	30(11.7)	6(10.9)	10(9.3)	14(14.1)	
severe depression	2(0.8)	0(0)	2(1.9)	0(0)	

ISI: Insomnia Severity Index, PSWQ: Penn State Worry Questionnaire, ZSDS: Zung Self-Rating Depression Scale

**Table 4:** Relationship between ISI, PSWQ and ZSDS with the Demographic Variables

**Variable**	**ISI**	**PSWQ**	**ZSDS**	**PPAQ**
Age(years)	NS	NS	NS	NS
Education	0.002	0.001	NS	NS
Primary	9.96(5.54)	46.02(9.89)		
Secondary	14.53(5.91)	55.49(13.84)		
University	12.7(5.4)	52.55(11.89)		
Number of previous deliveries	0.001	0.019	NS	NS
0	14.83(5.57)	55.64(11.14)		
1,2	12.12(5.49)	51.42(12.52)		
Job	NS	NS	0.04	NS
Worker			53.78(11.54)	
Housewives			49.00(12.32)	
Sufficiency of income for expenses	NS	NS	NS	NS
Sufficient				
Insufficient				
Abortion	NS	NS	NS	NS
No				
Yes				

NS = non-significant accusation, ISI: Insomnia Severity Index, PSWQ: Penn State Worry Questionnaire, ZSDS: Zung Self-Rating Depression Scale

**Table 5:** Multivariate Regression between ISI, PSWQ, ZSDS, PPAQ and Subscales of QOL

**Variable**	**ISI**		**PSWQ**		**ZSDS**		**PPAQ**	
	**F**	**P-value**	**F**	**P-value**	**F**	**P-value**	**F**	**P-value**
General QOL	32.279	<0.0001*	46.976	<0.0001*	2.84	0.093	0.714	0.399
General health	17.176	<0.00001*	11.132	0.001*	4.035	0.46	1.217	0.271
Physical health	22.327	<0.0001*	5.768	0.017*	10.009	0.002*	1.68	0.196
Psychological health	21.738	0.0001*	12.397	0.001*	7.449	0.007*	1.127	0.289
Social relationships	1.273	0.26	0.005	0.946	2.68	0.103	0.594	0.442
Environment	0.255	0.614	0.462	0.497	1.473	0226	1.687	0.195

QOL: quality of life, PA: Physical activity, ISI: Insomnia Severity Index, PSWQ: Penn State Worry Questionnaire, ZSDS: Zung Self-Rating Depression Scale, PPAQ: Pregnancy Physical activity questionnaire, WHOQOL: World Health Organization Quality Of Life

## Limitation

Although all the questionnaires used in this study had acceptable levels of validity and reliability, the answers were the result of women's own perceptions of insomnia, worry, depression, PA, and QoL, as the questionnaires were all self-administered. The design of the study and sampling method was another limitation of the study, because a cross sectional study presents a limited account for causal relationships, and in a convenience sampling method, not all the women had the same chance to participate. Future studies can use longitudinal methods to determine the causes of insomnia, depression, worry, and lack of physical activity in pregnant women.

## Conclusion

The results of this study clearly showed those factors that can significantly affect QoL in pregnancy. In this study, the prevalence of insomnia, worry, and depression in pregnant women was very high, and given the role of these 3 factors in QoL, women need to be informed about the necessity of controlling and reducing insomnia, worry, and depression to have a higher QoL. Moreover, in this study, it was found that PA declined during the second and third trimester of pregnancy, even though PA in pregnancy can positively impact general QoL.
